# Identification of Genetic Variants Associated with Sex-Specific Lung-Cancer Risk

**DOI:** 10.3390/cancers13246379

**Published:** 2021-12-20

**Authors:** Xiaoshun Shi, Sylvia Young, Grant Morahan

**Affiliations:** Harry Perkins Institute of Medical Research, QEII Medical Centre and Centre for Medical Research, The University of Western Australia, 6 Verdun Street, Nedlands, Perth, WA 6009, Australia; xiaoshun.shi@research.uwa.edu.au (X.S.); sylvia.young@uwa.edu.au (S.Y.)

**Keywords:** lung cancer, X chromosome, GWAS, sex-specific cancer susceptibility

## Abstract

**Simple Summary:**

The incidence of lung cancer differs between men and women, suggesting the potential role of sex-specific influences in susceptibility to this cancer. While behavioural differences, such as smoking rates, may account for much of the risk, another possibility is that X chromosome susceptibility genes may have an effect. Therefore, in this study, we tested specifically for the influence of X chromosome single-nucleotide polymorphisms (SNPs) in male lung cancer cases, and found 24 that were significantly associated with male, but not female, lung cancer cases. Examining these in detail, we observed these SNPs resided in blocks near the annotated genes *DMD*, *PTCHD1-AS*, and *AL008633.1*. We also observed that *DMD* was differentially expressed in lung cancer subtypes curated in the Cancer Genome Atlas database. Examining this gene further, we found that expression and mutation of DMD may have effects on immune function. This work defines potential targets for sex-specific lung cancer prevention.

**Abstract:**

Background: The incidence of lung cancer differs between men and women, suggesting the potential role of sex-specific influences in susceptibility to this cancer. While behavioural differences may account for some of the risk, another possibility is that X chromosome susceptibility genes may have an effect. Little is known about genetic variants on the X chromosome that contribute to sex-specific lung-cancer risk, so we investigated this in a previously characterized cohort. Methods: We conducted a genetic association reanalysis of 518 lung cancer patients and 844 controls to test for lung cancer susceptibility variants on the X chromosome. Annotated gene expression, co-expression analysis, pathway, and immune infiltration analyses were also performed. Results: 24 SNPs were identified as significantly associated with male, but not female, lung cancer cases. These resided in blocks near the annotated genes *DMD*, *PTCHD1-AS*, and *AL008633*.*1*. Of these, *DMD* was differentially expressed in lung cancer cases curated in The Cancer Genome Atlas. A functional enrichment and a KEGG pathway analysis of co-expressed genes revealed that differences in immune function could play a role in sex-specific susceptibility. Conclusions: Our analyses identified potential genetic variants associated with sex-specific lung cancer risk. Integrating GWAS and RNA-sequencing data revealed potential targets for lung cancer prevention.

## 1. Introduction

Consecutive epidemiological studies have found that the estimated new lung and bronchus case rate is higher in males than in females [1,2,3], suggesting that gender differences contribute to the incidence of lung cancer. Tobacco is a carcinogen that increases the risk of lung cancer. However, it is controversial whether the difference in lung cancer susceptibility in smokers is greater in women than in men compared with non-smokers [4,5]. Beyond smoking exposure, we hypothesized there could be a genetic effect on increased susceptibility in men. Many lung cancer susceptibility loci have been identified by genome-wide association studies (GWAS) [6]. X-linked genetic variants could affect susceptibility in males, but none of the reported SNPs were X-linked, because such associations were not specifically tested. Cancer sex disparity at the molecular level has been reported from somatic mutations in the TCGA database [7] and some studies proposed this disparity was associated with genes on the X chromosome [8,9]. However, these studies did not analyse germline variants, nor did they examine the sexes separately nor focused on somatic mutation, so did not consider the contribution of inherited X chromosome SNPs to gender differences in lung cancer.

Gender differences in susceptibility to various other types of cancer have been reported. A sex-stratified analysis of brain cancer GWAS data indicated that rs11979158 (7p11.2) was only associated with glioma in males [10]. In nasopharyngeal carcinoma, different genetic effects on males and females were revealed by studying susceptibility loci on the X chromosome [11]. Association analysis between genetic variants with obesity or colorectal cancer revealed that variants in the (autosomal) leptin gene harboured sex-specific associations with CRC risk [12]. Therefore, a deeper insight into lung cancer X chromosome SNPs could provide a better understanding of the genetic basis of sex predisposition difference. In this study, we performed an association analysis to test whether significant X chromosome SNPs were associated with lung cancer in men but not in women.

## 2. Materials and Methods

### 2.1. Data Accession and Categorization

The GWAS dataset in this analysis was downloaded with appropriate approvals from the dbGaP database (phs000093.v2. p2). A total of 513 lung cancer cases and 834 controls were retrieved based on phenotype document. The samples were subgrouped based on gender, resulting in 54 male SCLC cases, 27 female SCLC cases, 259 male NSCLC cases, and 173 female NSCLC cases. The age and family history groups in the 313 male lung cancer patients were defined based on the phenotype files in the dbGAP data set. We defined “younger age” as 64 or less (code 0–1) and “older age” as 65 or more (code 2–3). Twenty male patients had an incomplete family history, so only 293 patients were included for analysis of peak SNPs by family history. The genetic data were imputed by the fcGENE [13]. As part of our quality control procedure, we excluded samples and SNPs based on the following criteria: a. any SNP that had >5% heterozygous genotypes in all male samples; b. any male sample with >5% heterozygosity across all SNPs; c. SNPs in the pseudo-autosomal region (PAR) were removed; d. any designated female samples that were homozygous for >90% of the SNPs.

A list of pathogenic *DMD* SNPs was accessed from the Catalogue of Somatic Mutations in Cancer (COSMIC, https://cancer.sanger.ac.uk/cosmic) (accessed on 4 March 2021) and the Single Nucleotide Polymorphism Databases (dbSNP, https://www.ncbi.nlm.nih.gov/snp/) (accessed on 12 March 2021).

### 2.2. Association Analysis

The case against control association test on each subgroup, linkage disequilibrium (LD) analysis, haplotype analysis, and SNP annotation were conducted using Plink v1.07 with default settings. All significant SNPs were annotated using information from dbSNP (GRCh38.p12, https://www.ncbi.nlm.nih.gov/snp/) (accessed on 12 March 2021). Population-specific haplotype frequencies were analysed and visualized by LDhap (https://ldlink.nci.nih.gov/?tab=ldhap) (accessed on 25 April 2021) with reference to the “British in England and Scotland” and “Utah residents from North and West Europe” datasets.

### 2.3. Bioinformatics Analysis for DMD Expression and Mutation

We analysed the expression level of the *DMD* gene with associated clinicopathological features and obtained co-expressed genes in lung adenocarcinoma and squamous cell carcinoma of the lung by the UALCAN online tool (http://ualcan.path.uab.edu/index.html) (accessed on 25 February 2021) [14], visualizing by GEPIA2 (http://gepia2.cancer-pku.cn/#index) (accessed on 25 February 2021). [15]. The gene mutation profile data were analysed using the cBioportal (https://www.cbioportal.org/) (accessed on 25 February 2021). Survival data from microarray studies were accessed from PrognoScan (http://dna00.bio.kyutech.ac.jp/PrognoScan/index.html) (accessed on 26 February 2021). The relationship between immune infiltration and *DMD* mutations was analysed using TIMER2.0 (http://timer.cistrome.org/) (accessed on 25 February 2021).

### 2.4. GO/KEGG Pathway Enrichment Analysis

We applied the clusterProfiler package in R for the gene cluster analysis [16]. The unified positively co-expressed genes of *DMD* from both LUAD and LUSC were used for the Kyoto Encyclopedia of Genes and Genomes (KEGG) Gene Ontology (GO) enrichment analysis, including biological process (BP), cellular components (CC), and molecular function (MF). *p*-values < 0.05 were considered to indicate significantly enriched pathways.

### 2.5. Statistical Analysis

Fisher’s exact test was performed to calculate the significance of SNP genotype associations; the Kaplan–Meier method was used to estimate the impact of gene expression on survival; and P-values less than 0.05 were considered as statistically significant, except for GWAS SNP association, in which case, correction was made for testing of all the X chromosome SNPs.

## 3. Results

### 3.1. Identification of Sex-Specific SNPs Associated with Lung Cancer Susceptibility

**Figure 1:**
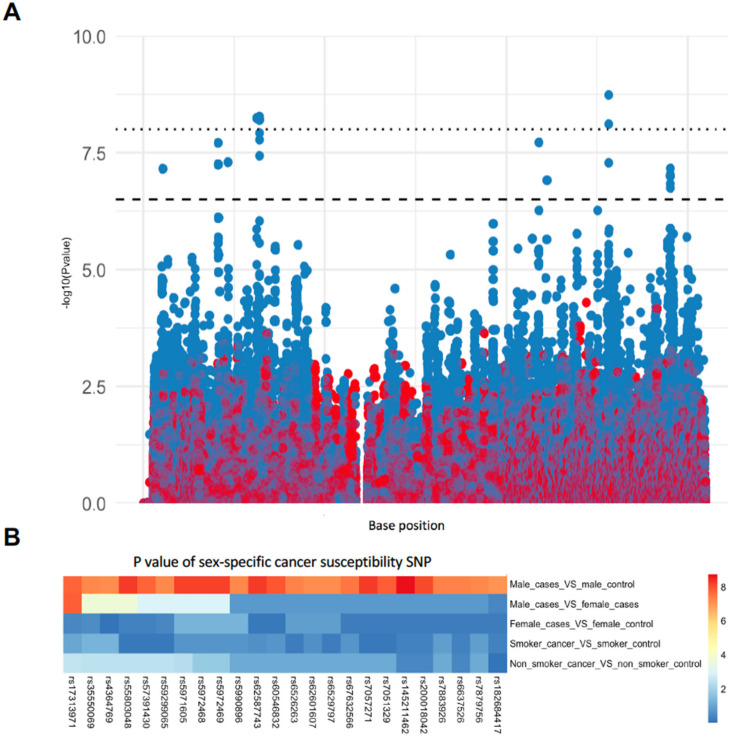
Identification of lung cancer risk sex-specific SNPs. (**A**) Manhattan plots of tested X chromosome SNPs. The distribution of the P-values of the X chromosome SNPs was plotted for the male (blue) and female (red) analyses. SNPs in the pseudo-autosomal regions, both PAR1 and PAR2, were not considered. The dashed line indicates the threshold for significant association as calculated by −log [0.05/171,804] where 171,804 was the number of X chromosome SNPs tested. The dotted line indicates the accepted threshold for analyses involving SNPs over the whole genome. (**B**) Heatmap showing p values of identified SNPs in subgroup association analysis. The cells in red indicate the SNPs whose *p*-values were significant.

To find potential X chromosome lung cancer susceptibility genes, we compared male lung cancer cases with male controls using data from a previously characterised cohort derived from the Environment and Genetics in Lung Cancer Etiology Study (EAGLE) [17] and the Prostate, Lung, Colon and Ovary Study (PLCO) [18] Cancer Screening Trial. Access to these data was approved via dbGAP (phs000093.v2.p2). We identified a total of 24 significantly associated SNPs (Figure 1A and Table 1); all of these were outside the pseudo-autonomous (PAR) region. The genotypes of the most strongly associated SNPs that were over-represented in male lung cancer cases were the C alleles of rs145211462 and rs62587743, suggesting that the alleles of these SNPs contributed significantly to lung cancer susceptibility in males (Fisher’s Exact Test, Table 2). The genes that are located nearest to these SNPs are *AL008633.1* and *DMD*.

We tested whether the peak SNPs were associated with cancer in females. As shown in Figure 1A, there were no significantly associated SNPs considering only female cases versus female controls. Since X chromosome SNPs are obligatory homozygote in males, we also considered homozygosity at these SNPs in females by excluding heterozygotes from the analyses. As shown in Table 3, there was no significant difference in females who were homozygous for the SNP alleles (Fisher’s Exact Test), suggesting that the alleles of these SNPs contributed specifically to susceptibility only in males. This does not exclude potential gene dosage effects. Next, we compared the *p* values of the 24 significant SNPs in males to other groups, including male lung cancer versus female lung cancer, smokers with lung cancer cases versus smokers without lung cancer, and non-smokers with lung cancer cases versus non-smokers without lung cancer. As shown in Figure 1B, the identified SNPs that contributed specifically to lung cancer susceptibility in males were not associated with smoking behaviour, which is a known cancer predisposition risk. Since males inherit X-linked alleles from their mothers, we reasoned that the X-linked male lung cancer risk SNPs would not be associated with disease in men with a family history of lung cancer. This was found to be the case (Table S1). We also asked whether these SNPs were associated with a later age of cancer onset. Some of the identified SNPs were weakly associated with a later age of diagnosis (Table 4), but these results should be confirmed in a larger cohort. Together, these results further supported the argument that X-linked cancer susceptibility genes contribute to lung cancer in males, regardless of smoking status and family history of lung cancer.

### 3.2. Interactions between X Chromosome SNPs in Male Lung Cancer Risk

Next, we performed a haplotype-trait association analysis on pairs of SNPs from each peak. Significant associations between SNPs in different peaks were found, suggesting that these SNPs defined chromosome regions that were associated with male-specific lung cancer susceptibility. SNP–SNP interaction analyses of the genotypes in the peak SNPs was performed. Based on the results of the Chi-squared test of the risk alleles of these SNPs, we observed that some of the risk alleles may have an additive effect on lung cancer risk. The odds of these risk alleles to lung cancer risk were compared between men and women. In two-by-two combination analysis, the risk allele combinations that were significant for male cancer were not detected in female cases (Table 5). Similarly, most three-by-three risk allele combinations contributing to the risk of male lung cancer were not found in female lung cancer cases (Table 6). This further supports the notion that the X-linked SNPs were associated with the risk of lung cancer in males.

### 3.3. Effect of Sex-Specific Lung Cancer Risk SNPs on DMD Expression

**Figure 2:**
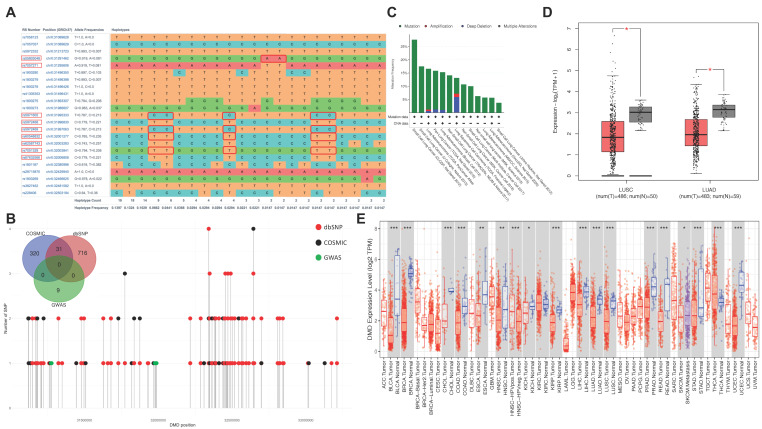
Genetic alteration and gene expression of DMD. (**A**) Haplotype analysis of identified SNPs in DMD. (**B**) Genomic position of identified SNPs, pathogenic SNPs in cancer and Duchenne muscular dystrophy in DMD. (**C**) The alteration profile of DMD in 3163 lung cancer samples reported in cBioportal. (**D**) Expression of DMD in TCGA-LUAD and TCGA-LUSC cohort. (**E**) Pan-cancer analysis of differential DMD expression. The significant p value was indicated as 0 ≤ *** < 0.001 ≤ ** < 0.01 ≤ * < 0.05.

The gene with the most annotated SNPs in this study was *DMD*, a very large gene that encodes the muscle protein, dystrophin. However, *PTCHD1-AS* and *AL008633.1*, the other two genes closely associated SNPs, were either not detected or not included in the relevant databases. Therefore, we focused on investigating the potential effect of *DMD* expression on lung cancer. We observed a haplotype pattern in these SNPs (Figure 2A), and their genomic position was close to SNPs identified as pathogenic in cancer and Duchenne muscular dystrophy (Figure 2B). The mutation profile in exons of the DMD gene in 3163 lung cancer samples was analysed in data from the cBioPortal for Cancer Genomics. A total of 14% of samples harboured *DMD* mutations, ranging from 3.75% to 27.59% in different cohorts (Figure 2C).

We next checked the gene expression of *DMD* in the TCGA—Lung adenocarcinoma (LUAD) and TCGA—Lung squamous cell carcinoma (LUSC) cohorts. The results showed that the mRNA expression levels of *DMD* were significantly decreased in the lung cancer tissues compared with the control tissues (Figure 2D). The differential expression of *DMD* between pan-cancer and corresponding control tissues was also investigated (Figure 2E), revealing that 55% (18 out of 33) of cancer types had abnormal DMD expression.

The impact of *DMD* on lung cancer survival was investigated, but its expression was not associated with either overall or disease-free survival in the lung cancer cohorts studied (Figure S1). We further analysed the effect of the differential expression of *DMD* on 1424 lung cancer patients in 13 microarray datasets (Table 7). We identified that *DMD* expression was associated with lung cancer survival in 4 out of 13 unified cohorts (30%), in which gene probes of different microarrays, such as 203881_s_at (GSE31210, *p* = 0.00004, relapse free survival of adenocarcinoma), A_24_P185854 (GSE13213, *p* = 0.00047, overall survival of adenocarcinoma), 203881_s_at (GSE31210, *p* = 0.00199061, overall survival of adenocarcinoma), 207660_at (GSE31210, *p* = 0.00427137, relapse-free survival of adenocarcinoma), 203881_s_at (jacob-00182-UM, *p* = 0.0116482, overall survival of adenocarcinoma), 234752_x_at (GSE8894,p= 0.0379615, and relapse-free survival of non-small cell lung cancer). This result suggested that *DMD* expression may play a minor role in lung cancer survival.

**Figure 3:**
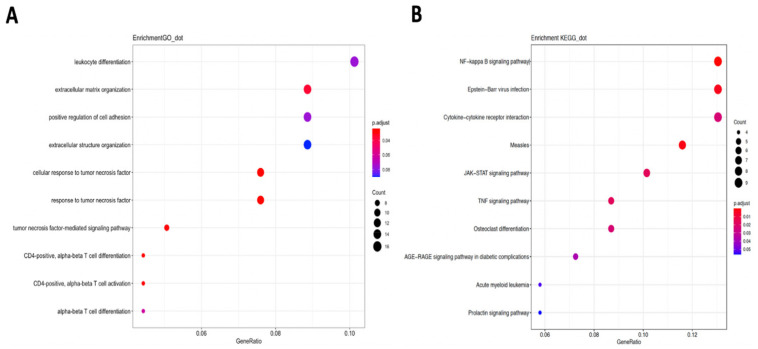
Gene ontology (GO) and KEGG pathway enrichment of DMD co-expressed genes in lung cancer. (**A**) All biological process enrichments of DMD co-expressed genes in lung cancer. (**B**) Kyoto Encyclopedia of Genes and Genomes analysis of the functional meanings of DMD co-expressed genes in lung cancer.

Genes that were co-expressed with *DMD* were identified by UALCAN online analysis [14]. A total of 5 genes in the TCGA-LUAD dataset and 180 genes in the TCGA-LUSC dataset with Spearman correlation coefficients greater than or equal to 0.4 were retrieved. No gene in either dataset was negatively co-expressed with *DMD* (with Pearson correlation coefficient <−0.3). We merged the positively co-expressed genes and performed in-silico analyses to explore the effects of expression *DMD* affected by X chromosome susceptibility SNPs in NSCLC. The enriched GO pathways for the co-expressed genes with *DMD* included “extracellular matrix organization” and “response to tumor necrosis factor” (Figure 3A), while the KEGG analysis implicated the NF−kappa B signaling pathway (Figure 3B).

### 3.4. DMD Could Affect CD4+ T Cell Infiltration in LUSC

**Figure 4:**
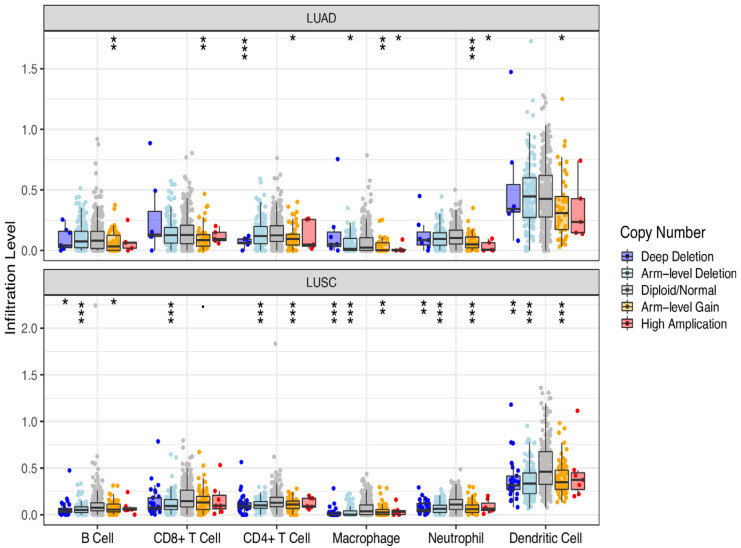
Correlation of DMD somatic copy-number alterations with immune infiltration levels in LUAD and LUSC. Box plots present the distributions of each immune subset based on each copy number status in LUAD and LUSC. The infiltration level for each SCNA category was compared with the control using a two-sided Wilcoxon rank-sum test. Significant p values are indicated as follows: 0 ≤ *** < 0.001 ≤ ** < 0.01 ≤ * < 0.05.

Copy number variation (CNV) has been observed in many studies to participate in the occurrence and development of cancer, and the number and complexity of CNVs are associated with the prognosis of many cancer types. Somatic copy-number alterations (SCNAs) affect a larger fraction of the genome, which can potentially activate an oncogene or inactivate a tumor suppressor gene. SCNAs can be further divided into focal SCNAs (shorter than one chromosome arm) and arm-level SCNAs (chromosome-arm length or longer) [19,20]. The SCNA subtypes, including deep deletion, arm-level deletion, diploid/normal, arm-level gain, and high amplification, can be defined by GISTIC 2.0 [20]. Studies of the correlation of gene mutation with immune infiltration levels in cancer facilitated the understanding of the interaction between malignant cells and the host immune system [21]. Therefore, we investigated the correlation of SCNA and tumor infiltration levels in LUAD and LUSC. As indicated in Figure 4, more tumor infiltrating cells were associated with *DMD* somatic copy-number alterations in LUSC than in LUAD. Of note, significant arm-level *DMD* deletion occurred in LUSC samples with CD4+ T cells infiltration (Figure 4), supporting the hypothesis that decreased expression of DMD caused by mutation may affect CD4+ T cell infiltration in LUSC.

## 4. Discussion

Genome-wide association testing is an important approach for the identification of genetic factors associated with complex genetic diseases such as lung cancer [6]. However, previous lung cancer GWA studies did not specifically test for potentially susceptible SNPs on the X chromosome. In this study, we performed an X chromosome-wide association study to identify susceptibility loci for lung cancer risk. We identified 24 significant SNPs in two X chromosomes that were associated with lung cancer in male patients. Based on the genome annotation, these SNPs mapped near the genes *DMD*, *PTCHD1-AS*, *and AL008633.1*.

Previous sex-specificity differences in lung cancer risk have been focused on tobacco-derived carcinogens, sex hormones, and carcinogen metabolism [22]. However, the intrinsic influence of genetic variants on sex-specific lung cancer risk should not be neglected. In the present study, we identified genetic variants on the X chromosome that were associated with lung cancer risk regardless of smoking, suggesting some male individuals who bear risk alleles of these X-linked genes are more susceptible to lung cancer. The synergistic interaction of SNPs could be associated with cancer susceptibility [23,24]. In this study, we identified that interactions between SNPs in different regions increase lung cancer risk. Further studies of genes in these regions could identify novel targets for lung cancer prevention.

*DMD* is a very large gene (greater than 2 Mb), and its mutations are known to be pathogenic in causing Duchenne and Becker muscular dystrophy. Recently, increasing evidence has suggested the role of *DMD* abnormality in cancer development. Leanne et al. summarized *DMD* mutations in major cancer types, including soft tissue sarcomas, tumours of the nervous system, carcinomas, and haematological malignancies [25]. Our study revealed that genetic variation in *DMD* (either as germline variants or as somatic mutations) could be associated with sex-specific risk of lung cancer. Consistent with previous findings, abnormal *DMD* expression was found in lung cancer compared with control tissues. However, the contribution of *DMD* to lung cancer susceptibility remains unclear. The pathway analysis of *DMD* co-expressed genes identified response to tumour necrosis factor in the GO and NF−kappa B signalling pathway in the KEGG pathways. Moreover, an association of the levels of immune infiltrates with *DMD* mutation was observed, suggesting that *DMD* may affect tumour development through abnormal immune processes. Altogether, *DMD* could be a molecular target for the prevention of some cases of male-specific lung cancer.

There are some limitations to this study. First, the dataset we used does not provide protein data, making a direct SNP–protein association analysis impossible. Second, the data were derived from subjects of European descent. Further investigation in other ethnic groups is needed. Third, it could be interesting to test whether these or other X-linked SNPs affected other cancer types. Fourth, collecting further DNA samples for X chromosome sequencing could validate our SNPs or provide novel SNPs associated with sex-specific lung cancer risk.

## 5. Conclusions

In this study, we performed analyses of GWAS data to identify sex-specific SNPs, located on chromosome X, associated with lung cancer. Based on gene annotation, expression analysis, co-expression analysis, and functional analyses, our findings support the hypothesis that DMD is abnormally expressed in cancer tissue and *DMD*-induced immune dysregulation may be responsible for the etiology of lung cancer. Further biomolecular experiments are needed to understand the interaction of these SNPs with *DMD*. Finally, it is well known that some simple single-gene diseases are more common in males due to inherited mutations in X-liked genes; our results may provide a paradigm for inherited X-linked variants contributing to susceptibility to other cancers and in other common complex genetic diseases.

## Figures and Tables

**Table 1:** Significant X chromosome SNPs associated with lung cancer in males.

SNP	BP	A1	F_A	F_U	A2	CHISQ	P *	OR	Gene
rs6529797	5454436	T	0.1709	0.08176	G	29.07	7 × 10^−8^	2.315	-
rs4364769	5462201	T	0.1709	0.08176	G	29.07	7 × 10^−8^	2.315	-
rs17313971	20597131	G	0.1262	0.04822	T	31.55	2 × 10^−8^	2.851	-
rs59299065	20600981	G	0.1182	0.04507	C	29.49	5.6 × 10^−8^	2.84	-
rs7879756	20601076	A	0.1182	0.04507	C	29.49	5.6 × 10^−8^	2.84	-
rs35550069	20602845	C	0.1182	0.04507	T	29.49	5.6 × 10^−8^	2.84	-
rs5990896	20603703	G	0.1182	0.04507	A	29.49	5.6 × 10^−8^	2.84	-
rs7883926	23285507	C	0	0.04612	A	29.7	5.1 × 10^−8^	0	*PTCHD1-AS*
rs6526263	23286674	G	0	0.04612	T	29.7	5.1 × 10^−8^	0	*PTCHD1-AS*
rs55803048	31273345	A	0.1821	0.08386	G	33.88	5.9 × 10^−9^	2.433	*DMD*
rs7057271	31277489	T	0.1837	0.08491	A	33.96	5.6 × 10^−9^	2.426	*DMD*
rs5971605	31977216	C	0.3035	0.1782	T	33.74	6.3 × 10^−9^	2.01	*DMD*
rs5972468	31978713	T	0.3035	0.1782	C	33.74	6.3 × 10^−9^	2.01	*DMD*
rs5972469	31978946	C	0.3035	0.1782	T	33.74	6.3 × 10^−9^	2.01	*DMD*
rs60546832	31983160	T	0.3035	0.1803	C	32.49	1.2 × 10^−8^	1.981	*DMD*
rs62587743	31985166	T	0.3514	0.218	C	34.06	5.3 × 10^−9^	1.943	*DMD*
rs7051329	31985724	T	0.2971	0.1761	G	31.84	1.7 × 10^−8^	1.978	*DMD*
rs67632566	31988691	T	0.2939	0.1761	C	30.31	3.7 × 10^−8^	1.948	*DMD*
rs57391430	109757735	G	0.2812	0.4203	A	31.59	1.9 × 10^−8^	0.5394	*-*
rs145211462	129041862	T	0.03834	0.1279	C	36.14	1.8 × 10^−9^	0.2719	*AL008633.1*
rs200018042	129048102	G	0.1565	0.282	C	33.34	7.7 × 10^−9^	0.4726	*AL008633.1*
rs6637526	129048867	G	0.2604	0.3931	A	29.64	5.2 × 10^−8^	0.5436	*AL008633.1*
rs182684417	146125695	G	0.1757	0.08595	A	28.53	9.2 × 10^−8^	2.267	-
rs62601607	146134454	T	0.1534	0.06918	C	29.1	6.9 × 10^−8^	2.437	-

* In this table, *p* values less than or equal to 9.2 × 10^−8^ were considered significant. SNP: single-nucleotide polymorphism ID; BP: physical base-pair position; A1: minor allele (based on whole sample); F_A: frequency of this allele in cases; F_U: frequency of this allele in controls; A2: major allele; CHISQ: basic allelic test chi-square (1df); P: asymptotic *p*-value for this test; OR: estimated odds ratio; Gene: annotated genes near the SNPs.

**Table 2:** Genotype of peak SNPs in males.

Peak SNPs	Genotype	Cases (*n* = 313)	Controls (*n* = 477)	OR *	*P*
rs6529797	T	54	39	1.56	<0.01
	G	259	438		
rs17313971	G	44	27	1.65	<0.01
	T	269	450		
rs7883926	A	313	455	NA	<0.01
	C	0	22		
rs55803048	A	57	40	1.59	<0.01
	G	256	437		
rs145211462	C	301	416	2.55	<0.01
	T	12	61		
rs62601607	T	48	33	1.59	<0.01
	C	265	444		

* OR: odds ratio; *P*: *p* value.

**Table 3:** Genotype of peak SNPs in females.

SNP	Genotype	Cases	Controls	OR *	*P*
rs6529797	T	51	71	1.14	0.31
	G	164	282		
rs17313971	G	25	39	1.04	0.83
	T	190	314		
rs7883926	A	202	321	1.34	0.19
	C	13	32		
rs55803048	A	47	69	1.09	0.51
	G	168	284		
rs145211462	C	189	304	1.11	0.54
	T	26	49		
rs62601607	T	47	74	1.03	0.8
	C	168	279		

* OR: odds ratio; *P*: *p* value.

**Table 4:** Genotype of peak SNPs by age.

SNP *	Age	Resistance Allele	Risk Allele	*P*
rs6529797	Under 65	134	19	0.04
	Over 65	125	35	
rs17313971	Under 65	129	24	0.52
	Over 65	140	20	
rs55803048	Under 65	129	24	0.31
	Over 65	127	33	
rs145211462	Under 65	10	143	0.02
	Over 65	2	158	
rs62601607	Under 65	125	28	0.16
	Over 65	140	20	

* The minor allele of rs7883926 was not present in any of the cases; therefore, this SNP was not included. Number of male cases is 313.

**Table 5:** Risk alleles pairwise combination analysis.

Combination *	Genotype	2 by 2 Risk Alleles Combination					
		Male			Female		
		Cancer	Control	Odds	Cancer	Control	Odds
X1_2	G_T	220	411	0.53	190	314	0.61
X1_3	T_A	54	39	1.38	None		
X1_4	T_A	12	2	6	None		
X1_5	T_C	52	31	1.68	None		
X1_6	T_T	5	2	2.5	None		
X2_3	G_A	44	26	1.70	None		
X2_4	G_A	10	2	5	None		
X2_5	G_C	42	26	1.62	None		
X2_6	G_T	6	2	3	None		
X3_4	A_A	57	39	1.46	47	69	0.68
X3_5	A_C	301	397	0.76	189	304	0.62
X3_6	A_T	48	31	1.55	47	74	0.64
X4_5	A_C	57	33	1.73	None		
X4_6	A_T	8	4	2	None		
X5_6	C_T	47	28	1.68	None		

* The risk alleles of representative SNPs in each peak were retrieved. The number of samples with the combination of these risk alleles were calculated. Each combination was labelled as “X risk allele in peak number_ allele in peak number”.

**Table 6:** Risk alleles three-by-three combination analysis.

Combination *	Genotype	Cancer	Control	Odds
X1_2_3	T_G_A	5	0	NA
X1_2_4	T_G_A	1	0	NA
X1_2_5	T_G_C	5	0	NA
X1_2_6	-	-	-	-
X1_3_4	T_A_A	12	2	6
X1_3_5	T_A_C	52	31	1.68
X1_3_6	T_A_T	52	31	2.5
X1_4_5	T_A_C	12	2	6
X1_4_6	-	-	-	-
X1_5_6	T_C_T	5	2	2.5
X2_3_4	G_A_A	5	2	5
X2_3_5	G_A_C	42	25	1.68
X2_3_6	G_A_T	6	2	3
X3_4_5	A_A_C	57	32	1.78
X3_4_6	-	-	-	-
X4_5_6	A_C_T	8	3	2.67

* The risk allele of representative SNPs in each peak were retrieved. Next, the number of samples with the combination of these risk alleles were calculated. Each combination was labelled as “X risk allele in peak number_ allele in peak number”. None of these combinations were identified in female.

**Table 7:** Survival analysis of DMD in lung cancer microarray cohorts.

Dataset	Lung Cancer Subtype	Endpoint	Probe ID	*N*	COX *p*-Value	HR,95% CI [Lower-Upper Bound] *
GSE31210	Adenocarcinoma	Relapse-free survival	203881_s_at	204	**<0.01**	0.46 [0.32–0.67]
GSE13213	Adenocarcinoma	Overall survival	A_24_P185854	117	**<0.01**	0.64 [0.50–0.82]
GSE31210	Adenocarcinoma	Overall survival	203881_s_at	204	**<0.01**	0.46 [0.29–0.75]
GSE31210	Adenocarcinoma	Relapse-free survival	207660_at	204	**<0.01**	0.53 [0.34–0.82]
jacob-00182-UM	Adenocarcinoma	Overall survival	203881_s_at	178	**0.01**	0.76 [0.61–0.94]
GSE8894	# NSCLC	Relapse-free survival	234752_x_at	138	**0.04**	0.17 [0.03–0.91]
GSE31210	Adenocarcinoma	Relapse-free survival	208086_s_at	204	0.06	0.62 [0.37–1.01]
GSE8894	NSCLC	Relapse-free survival	207660_at	138	0.08	0.00 [0.00–3.61]
GSE3141	NSCLC	Overall survival	203881_s_at	111	0.11	0.78 [0.58–1.06]
jacob-00182-MSK	Adenocarcinoma	Overall survival	203881_s_at	104	0.16	0.78 [0.55–1.10]
jacob-00182-CANDF	Adenocarcinoma	Overall survival	203881_s_at	82	0.16	0.75 [0.50–1.13]
GSE13213	Adenocarcinoma	Overall survival	A_24_P34186	117	0.17	0.76 [0.51–1.13]
GSE31210	Adenocarcinoma	Overall survival	208086_s_at	204	0.18	0.64 [0.33–1.23]
GSE3141	NSCLC	Overall survival	207660_at	111	0.20	0.76 [0.50–1.15]
jacob-00182-MSK	Adenocarcinoma	Overall survival	208086_s_at	104	0.22	0.82 [0.60–1.12]
GSE31210	Adenocarcinoma	Overall survival	207660_at	204	0.26	0.69 [0.36–1.32]
MICHIGAN-LC	Adenocarcinoma	Overall survival	M18533_at	86	0.28	0.76 [0.46–1.25]
jacob-00182-UM	Adenocarcinoma	Overall survival	207660_at	178	0.28	0.69 [0.35–1.35]
GSE17710	Squamous cell carcinoma	Relapse-free survival	26567	56	0.28	1.70 [0.65–4.47]
GSE17710	Squamous cell carcinoma	Overall survival	26567	56	0.31	1.69 [0.62–4.64]
GSE17710	Squamous cell carcinoma	Relapse-free survival	3354	56	0.32	1.53 [0.67–3.50]
GSE14814	NSCLC	Disease-specific survival	208086_s_at	90	0.35	2.10 [0.44–9.94]
jacob-00182-MSK	Adenocarcinoma	Overall survival	207660_at	104	0.36	0.62 [0.22–1.75]
GSE17710	Squamous cell carcinoma	Relapse-free survival	11043	56	0.37	1.15 [0.85–1.56]
jacob-00182-HLM	Adenocarcinoma	Overall survival	207660_at	79	0.38	0.64 [0.24–1.73]
GSE4573	Squamous cell carcinoma	Overall survival	208086_s_at	129	0.40	0.83 [0.55–1.28]
GSE17710	Squamous cell carcinoma	Overall survival	3354	56	0.45	1.39 [0.58–3.34]
GSE31210	Adenocarcinoma	Overall survival	234752_x_at	204	0.46	0.88 [0.63–1.24]
GSE3141	NSCLC	Overall survival	234752_x_at	111	0.47	1.07 [0.89–1.29]
MICHIGAN-LC	Adenocarcinoma	Overall survival	S81419_at	86	0.50	0.73 [0.29–1.84]
GSE17710	Squamous cell carcinoma	Overall survival	8673	56	0.53	0.80 [0.39–1.63]
jacob-00182-CANDF	Adenocarcinoma	Overall survival	207660_at	82	0.53	0.77 [0.35–1.74]
jacob-00182-HLM	Adenocarcinoma	Overall survival	203881_s_at	79	0.54	0.92 [0.69–1.21]
GSE4716-GPL3696	NSCLC	Overall survival	3336	50	0.54	0.80 [0.40–1.62]
GSE3141	NSCLC	Overall survival	208086_s_at	111	0.60	0.89 [0.58–1.37]
GSE4573	Squamous cell carcinoma	Overall survival	207660_at	129	0.61	1.17 [0.63–2.16]
GSE14814	NSCLC	Overall survival	208086_s_at	90	0.63	1.48 [0.30–7.36]
GSE17710	Squamous cell carcinoma	Relapse-free survival	8673	56	0.64	0.85 [0.41–1.73]
GSE17710	Squamous cell carcinoma	Overall survival	11043	56	0.67	1.06 [0.80–1.41]
GSE31210	Adenocarcinoma	Relapse-free survival	234752_x_at	204	0.68	0.94 [0.71–1.25]
GSE4573	Squamous cell carcinoma	Overall survival	203881_s_at	129	0.72	0.95 [0.72–1.25]
GSE8894	NSCLC	Relapse-free survival	208086_s_at	138	0.74	0.87 [0.38–1.99]
GSE14814	NSCLC	Disease-specific survival	203881_s_at	90	0.77	1.10 [0.58–2.07]
GSE14814	NSCLC	Overall survival	207660_at	90	0.82	0.88 [0.28–2.78]
GSE8894	NSCLC	Relapse-free survival	203881_s_at	138	0.87	0.99 [0.83–1.17]
GSE14814	NSCLC	Disease-specific survival	207660_at	90	0.93	1.06 [0.29–3.90]
jacob-00182-UM	Adenocarcinoma	Overall survival	208086_s_at	178	0.96	1.00 [0.83–1.20]
GSE14814	NSCLC	Overall survival	203881_s_at	90	0.96	1.01 [0.57–1.79]
jacob-00182-HLM	Adenocarcinoma	Overall survival	208086_s_at	79	0.99	1.00 [0.78–1.29]

* HR: hazard ratio, 95% CI (lower and upper bounds]) # NSCLC: non-small cell lung cancer.

## Data Availability

The GWAS dataset in this analysis was downloaded with appropriate approvals from the dbGaP database (phs000093.v2. p2). Other databases used in this study are listed in Materials and Methods section.

## Supplementary Materials

The following are available online at https://www.mdpi.com/article/10.3390/cancers13246379/s1. Figure S1: Impact of DMD on lung cancer overall or disease-free survival. Table S1: Genotype of peak SNPs by family history.
