# Supplementary material for: Identification of Genetic Variants Associated with Sex-Specific Lung-Cancer Risk
Source: Cancers (Basel). 2021 Dec 20;13(24):6379. doi: 10.3390/cancers13246379 (PMC8699314; doi:10.3390/cancers13246379)
Supplement: Supplementary file 1 [file cancers-13-06379-s001.zip › Table S1.pdf]

Table S1. Genotype of peak SNPs by family history

	Allele	no FH	FH	P
rs6529797	Major Allele	208	37	0.5
	Risk Allele	43	5	
rs17313971	Major Allele	213	36	1
	Risk Allele	38	6	
rs7883926	Minor Allele	0	0	NA
	Risk Allele	251	42	
rs55803048	Major Allele	206	32	0.39
	Risk Allele	45	10	
rs145211462	Minor Allele	10	1	1
	Risk Allele	241	41	
rs62601607	Major Allele	214	35	0.82
	Risk Allele	37	7	

*FH: Family history. Only 293 patients were included in this analysis, as 20 patients with incomplete family history.
