# Supplementary figures and images for: Identification of Genetic Variants Associated with Sex-Specific Lung-Cancer Risk
Source: Cancers (Basel). 2021 Dec 20;13(24):6379. doi: 10.3390/cancers13246379 (PMC8699314; doi:10.3390/cancers13246379)

## LUAD

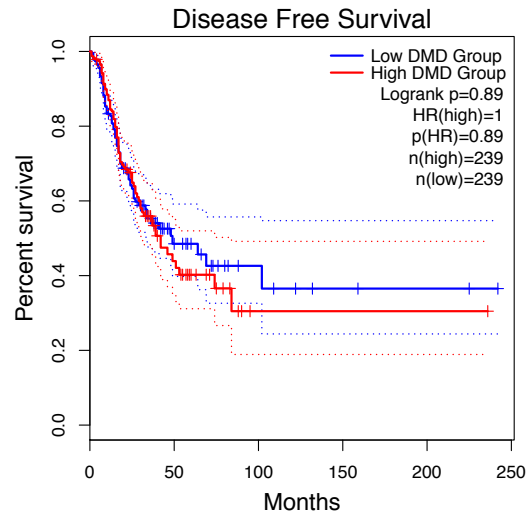

## LUSC

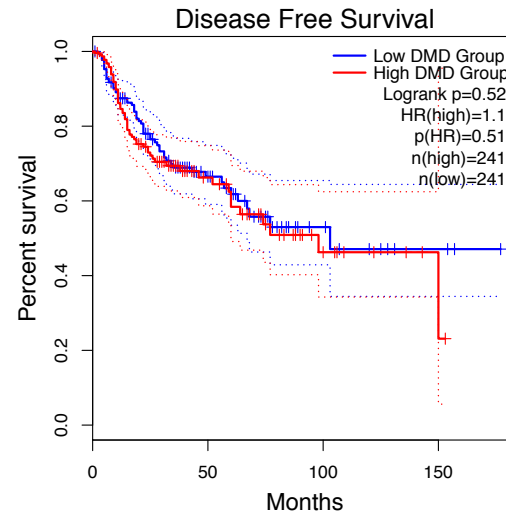

## LUAD+LUSC

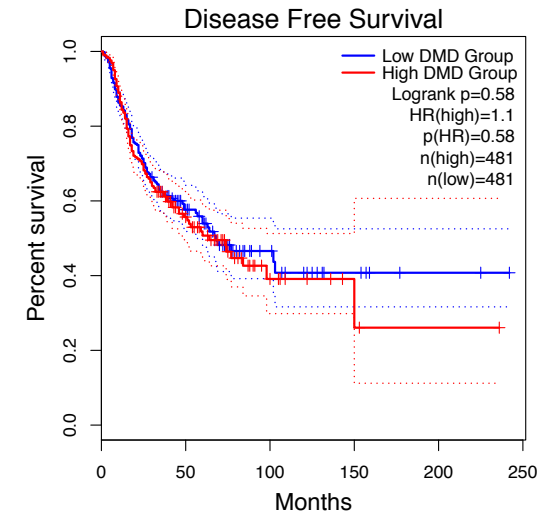

## Overall Survival

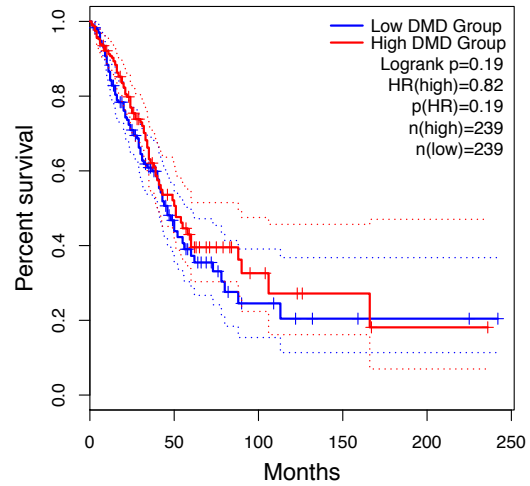

## Overall Survival

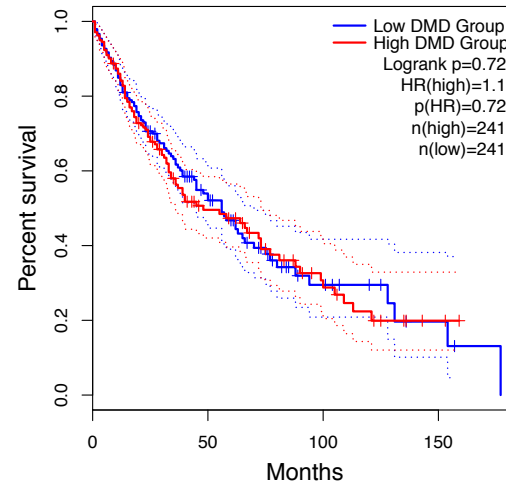

## Overall Survival

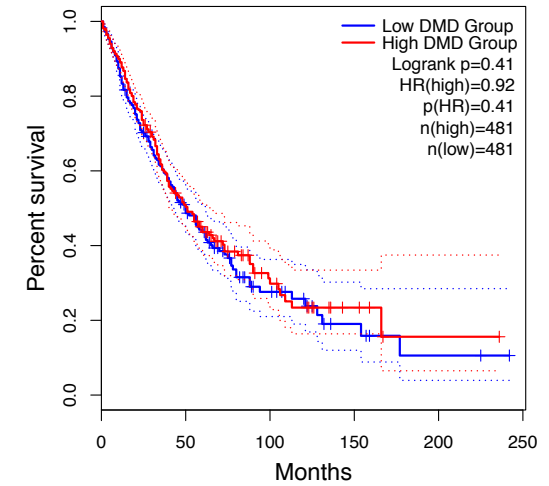

Supplement: Supplementary file 1 [file cancers-13-06379-s001.zip › Figure S1.pdf]
